# The performance of different classification criteria sets for spondyloarthritis in the worldwide ASAS-COMOSPA study

**DOI:** 10.1186/s13075-017-1281-5

**Published:** 2017-05-16

**Authors:** Pauline Bakker, Anna Moltó, Adrien Etcheto, Filip Van den Bosch, Robert Landewé, Floris van Gaalen, Maxime Dougados, Désirée van der Heijde

**Affiliations:** 1 Department of Rheumatology, Leiden University Medical Centre, P.O. Box 9600, 2300 RC Leiden, The Netherlands; 2 Department of Rheumatology, Paris Descartes University, Hôpital Cochin, Paris, France; 3 Inserm (U1153), Clinical Epidemiology and Biostatistics, Paris, France; 4 Department of Rheumatology, Ghent University Hospital, Ghent, Belgium; 5 Department of Rheumatology, Amsterdam Medical Centre, Amsterdam, The Netherlands; 6 Atrium Medical Centre, Heerlen, The Netherlands

**Keywords:** Spondyloarthritis, Classification criteria, Worldwide interregional differences

## Abstract

**Background:**

In this study, we sought to compare the performance of spondyloarthritis (SpA) classification criteria sets in an international SpA cohort with patients included from five continents around the world.

**Methods:**

Data from the (ASAS) COMOrbidities in SPondyloArthritis (ASAS-COMOSPA) study were used. ASAS-COMOSPA is a multinational, cross-sectional study with consecutive patients diagnosed with SpA by rheumatologists worldwide. Patients were classified according to the European Spondyloarthropathy Study Group (ESSG), modified European Spondyloarthropathy Study Group (mESSG), Amor, modified Amor, Assessment of SpondyloArthritis international Society (ASAS) axial Spondyloarthritis (axSpA), ASAS peripheral spondyloarthritis (pSpA) and ClASsification criteria for Psoriatic Arthritis (CASPAR) criteria. Overlap between the classification criteria sets was assessed for patients with and without back pain. Furthermore, patients fulfilling different arms of the ASAS axSpA criteria (imaging arm, clinical arm, both arms) were compared on the presence of SpA features.

**Results:**

A total of 3942 patients (5 continents, 26 countries) were included. The mean age was 43.6 years, 65.0% were male, 56.2% were human leucocyte antigen B27-positive and 64.4% had radiographic sacroiliitis (based on modified New York criteria). Of the patients, 85.5% were classified by the ASAS SpA criteria (87.7% ASAS axSpA, 12.3% ASAS pSpA). Fulfilment of the Amor, ESSG and CASPAR criteria was present in 83.3%, 88.4% and 21.6% of patients, respectively. Of the patients with back pain (*n* = 3227), most were classified by all three of Amor, ESSG and ASAS axSpA criteria (71.4%). Patients fulfilling the imaging arm and the clinical arm of the ASAS axSpA criteria had similar presentations of SpA features. In patients without back pain, overlap between classification criteria sets was seen, although to a lesser extent.

**Conclusions:**

Most patients with a clinical diagnosis of axial SpA in the worldwide ASAS-COMOSPA study fulfil several classification criteria sets, and a substantial overlap between different criteria sets is seen, which suggests a high level of credibility of the criteria. Large inter-regional differences in the fulfilment of classification criteria were not found. Patients fulfilling the clinical arm were remarkably similar to patients fulfilling the imaging arm with respect to the presence of most SpA features.

**Electronic supplementary material:**

The online version of this article (doi:10.1186/s13075-017-1281-5) contains supplementary material, which is available to authorized users.

## Background

Spondyloarthritis (SpA) encompasses a group of inter-related rheumatic conditions: ankylosing spondylitis (AS), including earlier forms of the disease that do not yet exhibit definitive structural damage on radiographs; psoriatic arthritis (PsA); arthritis associated with inflammatory bowel disease (IBD); and reactive arthritis [1]. Because SpA may have a heterogeneous presentation, a correct diagnosis is challenging. Rheumatologists make a diagnosis on the basis of what they have been taught during rheumatology training. The ‘art of diagnosing’ starts with a list of potential differential diagnoses, from among which the trained clinician deducts the most appropriate disease based upon the recognition of the ‘Gestalt’ and exclusion of other diagnoses.

Classification serves a completely different purpose, and several classification criteria sets of SpA are available. These classification criteria should be applied only in patients who have been diagnosed with SpA by a rheumatologist, and they cannot be used as a check box to be ticked in order to make the diagnosis. But the components of classification criteria may remind the clinician of the clinical picture of the disease. Different criteria sets put an emphasis on different features, and we do not know to what extent different criteria sets have penetrated different parts of the world. Therefore, we do not know which sets have influenced clinicians in particular regions most or to what extent these various sets of criteria describe more or less similar patients. Consequently, we do not know if rheumatologists around the world diagnose patients with a similar clinical picture of the disease.

The European Spondyloarthropathy Study Group (ESSG) criteria and the Amor criteria were developed to classify patients with SpA as a whole [2, 3]. In clinical practice, rheumatologists tend to distinguish patients with SpA according to their primary clinical presentation as patients with predominantly axial or predominantly peripheral complaints (with some overlap between these subtypes). The Assessment of SpondyloArthritis international Society (ASAS) has developed new criteria to better accommodate this distinction [4, 5]. These criteria sets can classify patients with predominantly axial symptoms as having axial spondyloarthritis (axSpA) and patients with predominantly peripheral symptoms as having peripheral spondyloarthritis (pSpA). The ASAS axSpA criteria consist of two arms: the imaging arm classifies patients who have sacroiliitis visualised on conventional radiographs and/or bone marrow oedema on magnetic resonance imaging (MRI), and the clinical arm classifies patients with normal imaging results. In 2006, a specific classification criteria set for PsA was developed, known as the ClASsification criteria for Psoriatic ARthritis (CASPAR) [6].

Classification criteria are used to include patients in clinical trials, cohort studies and other types of research. These criteria are frequently validated in restricted patient populations. We took the opportunity to investigate if rheumatologists worldwide diagnosed similar types of patients as having SpA by testing if patients fulfil similar criteria sets in the Assessment of SpondyloArthritis international Society COMOrbidities in SPondyloArthritis (ASAS-COMOSPA) study. Our assumption was that the more criteria sets a patient fulfils, the higher the likelihood that a patient with a diagnosis of SpA truly has SpA. The ASAS-COMOSPA study provides a unique opportunity to investigate this research question because it is, to our knowledge, the first observational study with such a large, worldwide population of patients with SpA, with axial and/or peripheral symptoms included [7].

## Methods

### Study population

The ASAS-COMOSPA study is an observational, cross-sectional, multicentre study which has been introduced elsewhere [7]. Participating rheumatologists were asked to include consecutive patients with a diagnosis of SpA from routine care. These patients had to fulfil the ASAS axSpA or pSpA criteria, but fulfilment of the ASAS criteria was not checked before inclusion. All information required to judge the fulfilment of various criteria sets, including the ASAS criteria, was collected in a random order (not grouped by criteria set) in the case report form.

Patients from 26 participating countries in 6 regions across the world (Western Europe, Central Europe, North America, Latin America, North Africa and Asia) were included. Western Europe was represented by Belgium, France, Germany, Hungary, Italy, the Netherlands, Portugal, Spain and the United Kingdom. Poland, Russia, Turkey and Ukraine were grouped into Central Europe. North America encompasses Canada and the United States, and Argentina, Brazil, Colombia and Mexico were summarized as Latin America. North Africa comprised Egypt and Morocco. China, Japan, Korea, Singapore and Taiwan were grouped and referred to as Asia. Approval by the local medical ethics committees, as well as written informed consent from all patients, was obtained before inclusion.

### Classification criteria

Patients were classified according to the following criteria sets: ESSG, Amor, ASAS SpA, ASAS axSpA, ASAS pSpA, imaging arm of ASAS axSpA, clinical arm of ASAS axSpA and CASPAR criteria [8]. The presence of either inflammatory back pain (IBP) or peripheral arthritis is a mandatory entry criterion of the ESSG criteria. According to the ESSG criteria, patients with at least one of the entry criteria in combination with one other minor criterion, such as enthesitis or psoriasis, are classified as having SpA [2]. Human leukocyte antigen B27 (HLA-B27) is *not* incorporated in this criteria set. The Amor criteria include a list of features with different weights, none of which is essential to classify a patient as having SpA, but a classification of SpA depends on the sum of weights [3]. Because patients in the COMOSPA study were not asked about the presence of balanitis, night pain and buttock pain, these items have not been taken into account, and therefore patients cannot collect points on these items in the Amor and ESSG criteria. The ESSG and Amor criteria were developed before MRI became widely available. In the present analysis, we also investigated the possibility of including inflammatory findings on MRI (ASAS definition [9]) as a feature in both the ESSG and Amor criteria, resulting in the modified Amor (mAmor) and modified European Spondyloarthropathy Study Group (mESSG) criteria.

The ASAS axSpA criteria consist of two arms, the imaging arm and the clinical arm, and can be applied only to patients with back pain of ≥3 months’ duration and an age of onset <45 years [10]. In patients with sacroiliitis visualised on pelvic radiographs or MRI, at least one other SpA feature should be present in order to be classified as axSpA according to the imaging arm [4]. In HLA-B27-positive patients, at least two other additional SpA features should be present in order to be classified as axSpA according to the clinical arm [4]. In patients without current back pain but with current peripheral manifestations, the classification for peripheral SpA can be applied. If a patient satisfies the entry criterion (current arthritis, enthesitis or dactylitis), the patient should have at least one other SpA feature if this is a specific SpA feature or at least two SpA features for less specific features [5]. Altogether, when current back pain (as defined above) is the presenting symptom, the ASAS axial SpA criteria should be applied. If arthritis/enthesitis/dactylitis is the presenting symptom, the peripheral SpA criteria should be applied. Together, these two sets form the ASAS SpA criteria.

A separate classification criteria set has been developed for PsA: the CASPAR criteria [6]. To meet the CASPAR criteria, the stem of the criteria demands first the presence of inflammatory articular disease and a score of at least 3 points derived from the presence of features such as skin psoriasis, dactylitis, nail lesions or juxta-articular bone formation visualised on radiographs (each feature is assigned a certain number of points). All above-described criteria sets are depicted in Additional file 1.

### Data analysis

Disease characteristics were described using descriptive statistics. The fulfilment of classification criteria was calculated for the cohort as a whole and thereafter per region. Subsequently, overlap between the different classification criteria was investigated and presented in Venn diagrams. This was done for patients with back pain and patients without back pain separately. Next, we looked in detail at the fulfilment of the ASAS axSpA criteria, comparing patients fulfilling only the clinical arm, patients fulfilling only the imaging arm and patients fulfilling both the clinical and imaging arms with regard to demographics and the presence of SpA features. Information on HLA-B27 must be available to be able to classify patients in the ‘imaging arm-only’ group, and information on imaging must be available to be able to classify patients in the ‘clinical arm-only’ group. IBM SPSS Statistics version 20.0 software (IBM, Armonk, NY, USA) was used for statistical analysis.

## Results

In total, 3984 patients were included in the COMOSPA study, with a mean number of SpA features of 5.5 (SD 1.8). The most common missing items were MRI of the sacroiliac joints (missing in 1951 patients), the presence of juxta-articular bone formation (missing in 999 patients) and HLA-B27 status (missing in 882 patients). There were 251 patients (6.4%) for whom both sacroiliac joint MRI and radiographs were not performed and 180 patients (4.6%) for whom HLA-B27 in addition was missing.

On the other hand, information on extra-articular manifestations was missing in none of the cases. Arbitrarily, a maximum of 6 missing items (total number of items 18) per patient was accepted. Patients with 7 or more missing items (*n* = 42) were left out of the analysis, which brings the total number of patients for this analysis to 3942. To define SpA features as present or absent, in order to apply the classification criteria, missing items were regarded as absent.

Demographics and disease characteristics are depicted in Table 1. Patients had a mean age of 44 years, and 65% were male. In the total cohort (patients with available data), HLA-B27 positivity was seen in 56% (73.0%) of the patients, and 54% (57.7%) had an elevated C-reactive protein level. Regarding the presence of sacroiliitis visualised on imaging, 64% (70.0%) presented with sacroiliitis seen on radiographs and 34% (94.8%) with sacroiliitis seen on MRI.

**Table 1:** Baseline characteristics

	Total patients (*n* = 3942)	Based on available data	Number of patients with missing items
Age at inclusion, years, mean ± SD	43.6 (13.9)		0
Male sex, *n* (%)	2563 (65.0%)		0
HLA-B27-positive, *n* (%)	2217 (56.2%)	72.0%	882
IBP, *n* (%)	3219 (81.7%)		52
Morning stiffness, *n* (%)	2497 (63.3%)		22
Enthesitis, *n* (%)	1354 (34.3%)		0
Dactylitis, *n* (%)	610 (15.5%)		3
Psoriasis, *n* (%)	843 (21.4%)		0
Uveitis, *n* (%)	771 (19.6%)		0
Peripheral arthritis, *n* (%)	2424 (61.5%)		0
IBD, *n* (%)	209 (5.3%)		0
Positive family history, *n* (%)	1475 (37.4%)		117
Good response to NSAIDs, *n* (%)	2433 (61.7%)	77.5%	803
Elevated CRP, *n* (%)	2109 (53.5%)	57.7%	287
Preceding infection, *n* (%)	271 (6.9%)		74
Sacroiliitis based on radiograph (mNY), *n* (%)	2539 (64.4%)	70.0%	341
Sacroiliitis based on MRI, *n* (%)	1326 (33.6%)	65.7%	1951
Rheumatoid factor-negative, *n* (%)	3177 (80.6%)	94.8%	613
Psoriatic nail dystrophy, *n* (%)	460 (11.7%)		28
Juxta-articular bone formation, *n* (%)	526 (13.3%)	17.7%	999

*Abbreviations: IBP* Inflammatory back pain according to Assessment of SpondyloArthritis international Society definition (reference), *IBD* Inflammatory bowel disease, *CRP* C-reactive protein, *ESR* Erythrocyte sedimentation rate, *HLA-B27* Human leucocyte antigen, *mNY* Modified New York criteria, *NSAID* Non-steroidal anti-inflammatory drug, *MRI* Magnetic resonance imaging

### Fulfilment of classification criteria

Most (92.6%) of the 3942 patients fulfilled the mESSG criteria. Fulfilment of Amor, mAmor, ESSG and ASAS criteria was all above 80% (Table 2). A minority (12.3%) of the patients fulfilled the ASAS pSpA criteria, whereas 21.6% of the patients fulfilled the CASPAR criteria. We emphasise that the criteria were applied to all patients; only the patients with seven or more missing values were left out, and missing items were regarded as absent.

**Table 2:** Fulfilment of classification criteria

Classification criteria	Patients who fulfilled the classification criteria, *n* (%)
Amor	3282 (83.3%)
mAmor	3454 (87.6%)
ESSG	3485 (88.4%)
mESSG	3652 (92.6%)
ASAS SpA total	3370 (85.5%)
ASAS axial SpA, current back pain	2955 (87.7%)
Both arms (imaging & clinical)	1737 (58.8%)
mNY+	976 (56.2%)
MRI+	169 (9.7%)
Both	592 (34.1%)
Imaging arm only	984 (33.3%)
mNY+	539 (54.8%)
MRI+	245 (24.9%)
Both	200 (20.3%)
Clinical arm only	234 (7.9%)
ASAS peripheral SpA	415 (12.3%)
CASPAR	852 (21.6%)

*Abbreviations: mAmor* Modified Amor, *ESSG* European Spondylarthropathy Study Group, *mESSG* Modified European Spondyloarthropathy Study Group, *ASAS* Assessment of SpondyloArthritis international Society, *mNY* Modified New York, *MRI* Magnetic resonance imaging, *CASPAR* ClASsification criteria for Psoriatic ARthritis

Most patients (*n* = 1507) were included in Western Europe (85 centres from 26 countries), followed by 1073 patients in Asia, 438 patients in Central Europe, 337 patients in Latin America, 337 patients in North Africa and 239 patients in North America. Regional differences in fulfilment of classification criteria are depicted in Table 3. When we looked in detail at the ASAS SpA criteria, we found that in Central Europe, 84% of the patients fulfilled the ASAS axial SpA criteria (ASAS peripheral criteria 5.3%), whereas in contrast, in North America, 51% of the patients fulfilled the axial SpA criteria (ASAS peripheral criteria 22.6%). In both Asia and Central Europe, a small minority of the patients fulfilled the ASAS pSpA criteria, and the axial complaints were by far the predominant symptoms. A relatively high percentage of patients fulfilled the CASPAR criteria in North America compared with the other regions. Less pronounced regional differences were seen regarding criteria sets that cover the whole spectrum of SpA, namely the Amor and ESSG criteria.

**Table 3:** Regional differences in fulfilment of classification criteria

	Number of patients included	Patients fulfilling Amor criteria, *n* (%)	Patients fulfilling mAmor criteria, *n* (%)	Patients fulfilling ESSG criteria, *n* (%)	Patients fulfilling mESSG criteria, *n* (%)	Total patients fulfilling ASAS SpA criteria, *n* (%)	Patients fulfilling ASAS axSpAcriteria, *n* (%)	Patients fulfilling, ASAS pSpA criteria, *n* (%)	Patients fulfilling CASPAR criteria, *n* (%)
Western Europe	1507	1242 (82.4%)	1333 (88.5%)	1335 (88.6%)	1433 (95.1%)	1305 (86.6%)	1149 (76.2%)	156 (10.4%)	388 (25.7%)
Central Europe	438	342 (78.1%)	364 (83.1%)	402 (91.8%)	419 (95.7%)	391 (89.3%)	368 (84.0%)	23 (5.3%)	32 (7.3%)
North America	239	203 (84.9%)	205 (85.8%)	219 (91.6%)	219 (91.6%)	176 (73.6%)	122 (51.0%)	54 (22.6%)	120 (50.2%)
Latin America	348	290 (83.3%)	307 (88.2%)	314 (90.2%)	326 (93.7%)	280 (80.5%)	239 (68.7%)	41 (11.8%)	93 (26.7%)
North Africa	337	300 (89.0%)	310 (92.0%)	316 (93.8%)	324 (96.1%)	308 (91.4%)	260 (77.2%)	48 (14.2%)	94 (27.9%)
Asia	1073	905 (84.3%)	935 (87.1%)	899 (83.8%)	931 (86.8%)	910 (84.8%)	817 (76.1%)	93 (8.7%)	125 (11.6%)

*Abbreviations: ASAS* Assessment of SpondyloArthritis international Society, *axSpA* Axial spondyloarthritis, *CASPAR* ClASsification criteria for Psoriatic Arthritis, *ESSG* European Spondyloarthropathy Study Group, *mAmor* Modified Amor, *mESSG* Modified European Spondyloarthropathy Study Group, *pSpA* Peripheral spondyloarthritis, *SpA* Spondyloarthritis

Percentages relate to number of patients of that specific region

### Overlap in classification criteria

**Fig. 1:**
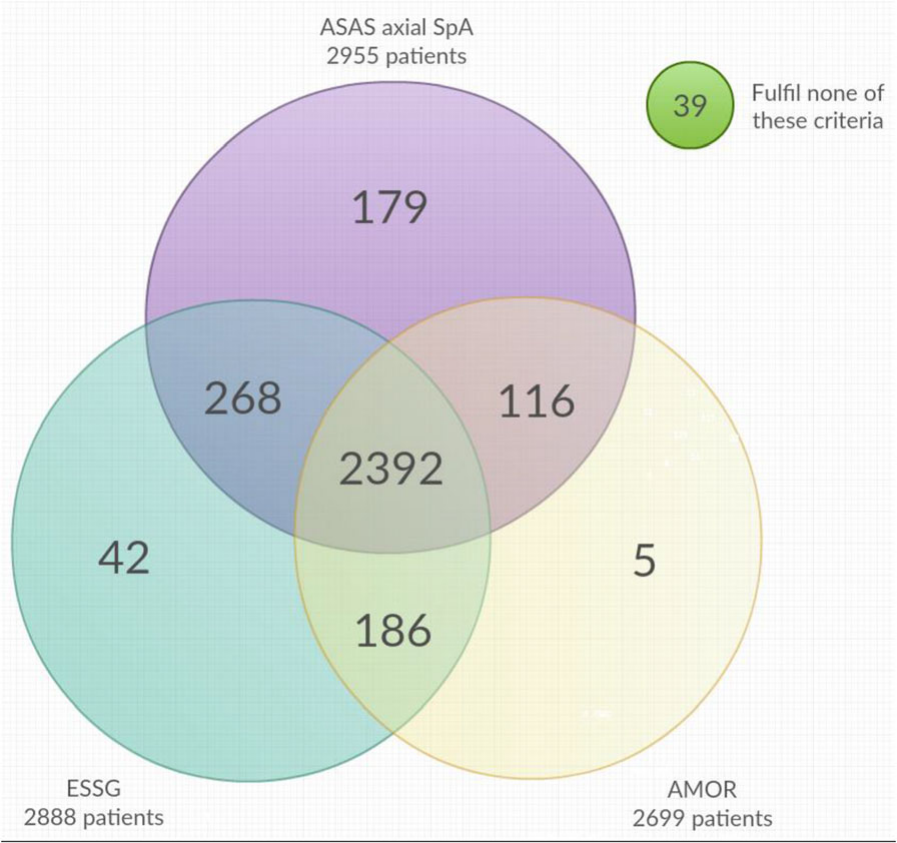
Overlap between ESSG, AMOR, and axSpA criteria in patients with current back pain (*n* = 3227)

**Fig. 2:**
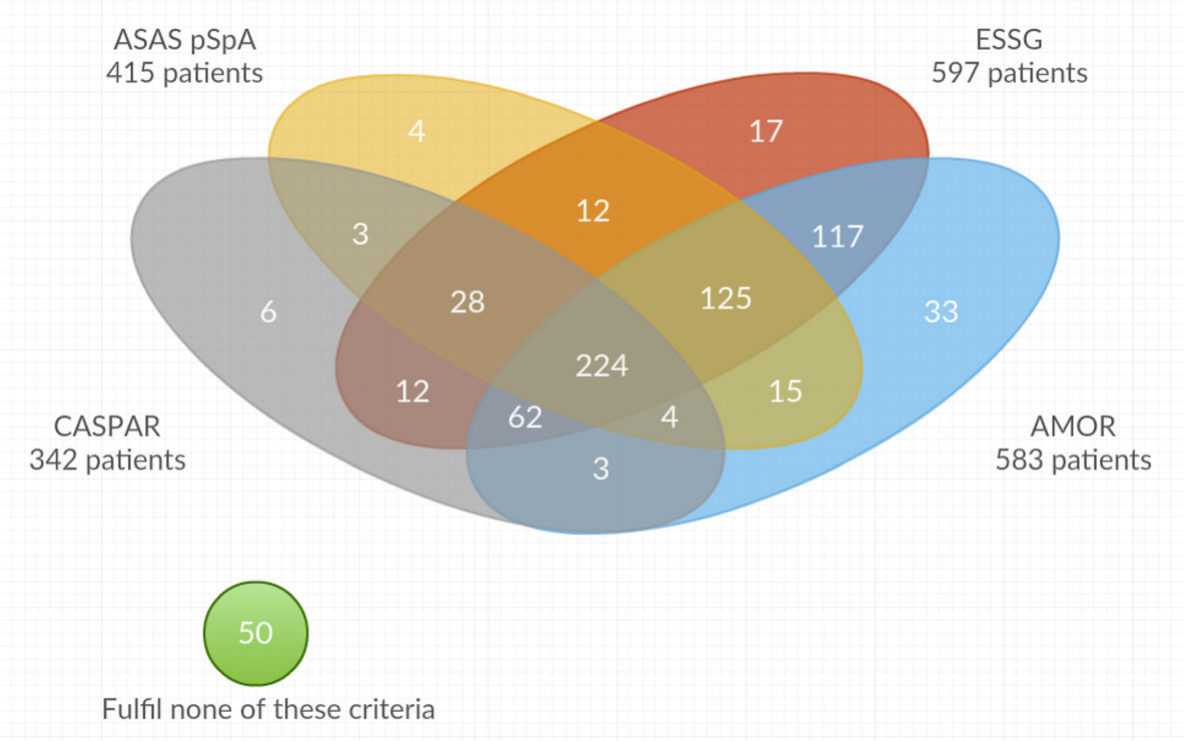
Overlap between the ESSG, AMOR, CASPAR, and pSpA criteria in patients without current back pain (*n* = 715)

Venn diagrams representing the overlap between the different criteria sets are shown in Figs. 1 and 2. Figure 1 reveals that the majority of the patients with back pain were classified by all three criteria sets: Amor, ESSG and ASAS axSpA criteria (*n* = 2392 [74.1%]). Most patients who fulfilled two criteria sets fulfilled both the ASAS axSpA criteria and the ESSG criteria (*n* = 268 [8.3%]). Few patients fulfilled only one criteria set. Most of the patients who were picked up by one criteria set only were classified by the ASAS axSpA criteria (*n* = 179 [5.5%] compared with 1.3% by the ESSG criteria only and 0.2% by the Amor criteria only). The major overlap of the criteria points to the typical clinical pattern of SpA the included patients have.

Regarding the patients without current back pain (peripheral complaints), again substantial overlap between the criteria was seen (ASAS pSpA, Amor, ESSG, CASPAR) (Fig. 2). Most of the patients fulfilled all four criteria sets (*n* = 224 [31.3%]). Subsequently, 125 patients (17.5%) fulfilled all criteria, except those for PsA-specific CASPAR criteria, which is not surprising, because the CASPAR criteria are focussed on the clinical disease PsA and not on other forms of pSpA. Only six patients (0.8%) fulfilled only the CASPAR criteria, and only four patients (0.6%) fulfilled only the ASAS pSpA criteria. Regarding overlap between the different criteria sets in the different regions, the same trends were seen, and no substantial inter-regional differences were found (data not shown).

### Comparison between patients fulfilling the ASAS imaging arm split by presence of HLA-B27 and the clinical arm only

Disease characteristics of patients fulfilling the imaging arm or the clinical arm only are depicted in Table 4. In addition, characteristics of patients fulfilling the imaging arm are presented on the basis of the presence or absence of HLA-B27. Only patients who have data available on HLA-B27 and imaging are included in Table 4. There were more male patients in the HLA-B27-positive imaging arm (74.1%) than in the HLA-B27-negative imaging arm (50.4%) and the clinical arm (53.1%). Psoriasis was seen more frequently in the group of HLA-B27-negative patients fulfilling the imaging arm. On the contrary, enthesitis and dactylitis were relatively more common in the patients who fulfilled only the clinical arm. A positive family history was also more frequently seen in the clinical arm than in the imaging arm (independent of HLA-B27 status).

**Table 4:** Comparison between the imaging arm and clinical arm of the ASAS axSpA criteria, with the required presence of HLA-B27 and imaging

	Imaging arm (%), HLA-B27 always available		Clinical arm alone (%), imaging always available (*n* = 98)
	HLA-B27-positive (*n* = 1746)	HLA-B27-negative (*n* = 546)	
Age, years	40.3 (13.0)	42.7 (13.0)	42.8 (13.0)
Male sex	1294 (74.1%)	275 (50.4%)	52 (53.1)
IBP	1639 (93.3%)	529 (96.9%)	87 (88.8%)
Peripheral arthritis	904 (51.8%)	302 (55.3%)	57 (58.2%)
Psoriasis	147 (8.4%)	105 (19.2%)	14 (14.3%)
Uveitis	453 (25.9%)	49 (9.0%)	27 (27.6%)
Enthesitis	563 (32.2%)	164 (30.0%)	44 (44.9%)
Dactylitis	137 (7.8%)	55 (10.1%)	17 (17.3%)
IBD	73 (4.2%)	46 (8.4%)	6 (6.1%)
Positive family history	669 (38.3%)	166 (30.4%)	52 (53.1%)
Good response to NSAIDs	1180 (67.6%)	265 (48.5%)	64 (65.3%)
Elevated CRP	1094 (62.7%)	256 (46.9%)	41 (41.8%)

*Abbreviations: HLA-B27* Human leucocyte antigen B27, *IBP* Inflammatory back pain, *IBD* Inflammatory bowel disease, *NSAID* Non-steroidal anti-inflammatory drug, *CRP* C-reactive protein

## Discussion

Appropriate diagnostic criteria for axSpA and pSpA do not exist and, in the absence of an unequivocal gold standard, will never be developed, but various classification criteria are available. These classification criteria have in common that they have been developed using the external standard ‘expert opinion’. But expert opinion is not an equivocal and homogeneous construct and may potentially integrate different pictures of the disease SpA.

The present study reveals that, in our cohort, most patients diagnosed as having SpA fulfilled multiple classification criteria sets, which adds to the credibility of the construct of SpA as a recognizable entity. Although the substantial overlap between the different criteria sets for patients with both axial and with peripheral symptoms could be expected, the fact that different criteria sets have been developed for different target populations (e.g., the ESSG, focussed on the whole concept of SpA; the ASAS axial SpA criteria for patients with SpA axial symptoms) could have precluded overlap in different regions of the world. In the present study, we have shown that the significant overlap was consistent all over the world, thus suggesting that rheumatologists worldwide use similar ‘pictures’ of what SpA is. In other words, they operationalise the construct of SpA approximately similarly. In addition, the huge overlap (e.g., 74.1% of the patients fulfilled all three criteria sets, and only 7.6% fulfilled one set only) confirms that the criteria for SpA are highly credible.

As mentioned already, large interregional differences in the fulfilment of classification criteria were not found. This is remarkable in the light of all genetic and environmental differences, as well as differences in resources and health care systems around the world. In fact, it appears that the clinical picture—and consequently the diagnosis—of SpA is remarkably homogeneous around the world, despite all possible differences in, for example, genetic background, prevalence and medical training.

Of course, there were some notable differences. The most important one was that more patients with PsA and fewer patients with axial disease were included in North America than in other regions. We do not think this reflects a true difference in the prevalence of the different subtypes of the disease. This is supported by a recent systematic review that pooled population prevalence estimates for SpA, AS and PsA in geographic areas [11]. The prevalence of both the axial and peripheral subtypes was, on average, comparable in North America to other parts of the world. More likely, the difference could be due to local factors, such as a difference in areas of interest of the doctors including patients or referral centres for a certain disease. One reason may be the perception of PsA as belonging to SpA or not. It is well known that some rheumatologists view PsA as a separate entity and others view PsA as a subtype of SpA. Apparently, more doctors in North America than in other parts of the world consider patients with PsA as having SpA.

Regarding the inclusion criteria of the study, doctors were required to include patients with SpA only if they thought the patient would fulfil the ASAS SpA criteria (either peripheral or axial). However, fulfilment of the ASAS criteria was not formally checked before inclusion, as described in the Methods section above. When analysing the data, it became clear that only 85.5% of the patients actually did fulfil the ASAS SpA criteria, ranging from 73.6% in North America to 91.4% in North Africa. This implies that the large majority of patients with SpA are indeed covered by the criteria, pointing to high sensitivity but also indicating that doctors diagnose SpA in patients who do not fulfil the ASAS criteria. However, we would like to make a critical comment which relates to a limitation of the present study. The fact that rheumatologists were initially asked to include ASAS SpA patients (although fulfilment of the ASAS criteria was not met in all patients) could very well have led to an ‘a priori’ high percentage of ASAS classification criteria fulfilment. This could have led to an overestimation of performance of sensitivity of the criteria.

The ASAS classification criteria were developed in recent history. The criteria were validated in an international study of more than 600 patients with chronic back pain of unknown origin. In the ASAS study population, the ASAS criteria compared favourably with other previously established criteria sets with regard to sensitivity and specificity. In our study, if patients with axial symptoms were picked up by one criteria set only, of all sets tested, the ASAS axSpA criteria were most sensitive (although the others performed well, too). The latter could be due to the fact that the ASAS-COMOSPA study is not a cohort of early disease (as reflected by 65% modified New York criteria positivity). Prior studies have shown that performances of, for example, ESSG and Amor criteria in early disease were (slightly) worse than the ASAS criteria [12]. A more likely explanation is that the rheumatologists were asked to include patients fulfilling the ASAS criteria.

Although the imaging arm of the ASAS classification criteria is broadly recognized as highly specific, there has been debate on the validity of the clinical arm of the ASAS criteria, which has not been well received by different national and international health care systems. In the literature, it has been argued that patients fulfilling only the clinical arm of the ASAS axSpA criteria should not be considered as having ‘true axSpA’. A reason why the clinical arm of the ASAS axSpA criteria has been developed is that MRI is not universally available. In our cohort, in which a large proportion of patients did not undergo MRI, our results demonstrate the value of the clinical arm of the ASAS criteria for scientific research. We found that patients fulfilling the clinical arm were remarkably similar to patients fulfilling the imaging arm with respect to the presence of many SpA features.

Strengths of the study are the multi-national cohort and the large number of patients included, which is unique, to our knowledge. Unfortunately, no control group was available, and therefore true specificity of the different classification criteria sets could not be calculated. Another limitation of the study is the relatively high number of missing values, especially when it comes to key items such as HLA-B27 and MRI. Unfortunately, this is a direct consequence of normal clinical practice: If sufficient information has been collected to make a diagnosis, further testing is often not performed (e.g., to save expenses).

We can conclude that, despite the heterogeneous character and varying prevalence of SpA as a disease across the world, similar patients are identified as having SpA by rheumatologists worldwide. Moreover, patients with the diagnosis of SpA usually fulfil multiple criteria sets, providing validity to the criteria, including the relatively new ASAS SpA criteria as well as to the concept of SpA. We emphasize that classification criteria for SpA were developed for use in epidemiological and clinical research and are not suitable for use as diagnostic tools in clinical practice.

## Conclusions

Most patients diagnosed with SpA by rheumatologists in five continents across the world fulfilled multiple classification criteria sets. To our knowledge, this is the first study confirming the validity of the ASAS axSpA criteria in a large, worldwide population of patients. Patients fulfilling the clinical and/or imaging arms of the ASAS axSpA criteria have comparable SpA features.

For the first time, to our knowledge, the performance of the various SpA classification criteria sets is assessed in a worldwide setting with a very large number of patients included from five different continents. We also took the opportunity to phenotypically compare patients fulfilling the different arms of the ASAS axial SpA criteria in terms of demographics and presence of SpA features, among others.

## Additional files

Additional file 1: Overview of different criteria sets used in this study. (DOCX 22 kb)

Additional file 2: Ethical bodies that approved the study in the various centres. (DOCX 14 kb)

## Abbreviations

AS: Ankylosing spondylitis
ASAS: Assessment of SpondyloArthritis international Society
axSpA: Axial spondyloarthritis
CASPAR: ClASsification criteria for Psoriatic Arthritis
COMOSPA: COMOrbidities in SPondyloArthritis
ESSG: European Spondyloarthropathy Study Group
HLA-B27: Human leucocyte antigen B27
IBD: Inflammatory bowel disease
IBP: Inflammatory back pain
mAmor: Modified Amor
mESSG: Modified European Spondyloarthropathy Study Group
mNY: Modified New York criteria
MRI: Magnetic resonance imaging
NSAID: Non-steroidal anti-inflammatory drug
PsA: Psoriatic arthritis
pSpA: Peripheral spondyloarthritis
SpA: Spondyloarthritis
