# Supplementary material for: The performance of different classification criteria sets for spondyloarthritis in the worldwide ASAS-COMOSPA study
Source: Arthritis Res Ther. 2017 May 16;19:96. doi: 10.1186/s13075-017-1281-5 (PMC5434574; doi:10.1186/s13075-017-1281-5)
Supplement: Supplementary file 1 — Overview of different criteria sets used in this study. (DOCX 22 kb) [file 13075_2017_1281_MOESM1_ESM.docx]

Additional file 1: Overview of different criteria sets used in this study

- Amor criteria (figure 1)

Modified Amor criteria (not shown)

*Rudwaleit M, van der Heijde D, Landewe R et al. The development of Assessment of SpondyloArthritis international Society classification criteria for axial spondyloarthritis (part II): validation and final selection. Ann Rheum Dis 68; 777-83.*

- ESSG criteria (figure 2)

Modified Amor criteria (not shown)

*Rudwaleit M, van der Heijde D, Landewe R et al. The development of Assessment of SpondyloArthritis international Society classification criteria for axial spondyloarthritis (part II): validation and final selection. Ann Rheum Dis 68; 777-83.*

- ASAS SpA criteria (figure 3)
- CASPAR criteria (figure 4)

Figure 1:

| **Amor criteria*** | |
| --- | --- |
| Items | Score |
| 1. **Clinical symptoms/history** |  |
| 1. Pain at night (spine) or morning stiffness | 1 |
| 1. Asymmetrical oligoarthritis | 2 |
| 1. Gluteal (buttock) pain (any) or alternating gluteal pain | 1 or 2 |
| 1. Sausage like digit or toe (dactylitis) | 2 |
| 1. Enthesitis (heel) | 2 |
| 1. Uveitis | 2 |
| 1. Urethritis/cervicitis within 1 month before onset of arthritis | 1 |
| 1. Diarrheae within 1 month before onset of arthritis | 1 |
| 1. Psoriasis, balanitis or inflammatory bowel disease | 1 |
| 1. **X-rays** |  |
| 1. Sacroiliitis (grade 2 bilaterally or grade 3 unilaterally) | 3 |
| 1. **Genetical background** |  |
| 1. HLA-B27 positive or positive family history for AS, ReA, uveitis, psoriasis or inflammatory bowel disease | 2 |
| 1. **Good response to NSAIDs** |  |
| 1. NSAIDs show a good response within 48 hours, or relapse within 48 hours after NSAIDs are stopped | 2 |
|  | ***At least 6 points are necessary*** |

**Amor B, Dougados M, Mijiyawa M [Criteria of the classification of spondylarthropathies]. Rev Rhum Mal Osteoartic 57; 85-9.*

Figure 2:

| **European Spondyloarthropathy Study Group (ESSG) criteria*** | | |
| --- | --- | --- |
| **Inflammatory back pain** | **OR** | **Synovitis**   - Asymmetric or - Predominantly in the lower limbs |
|  | **plus** one of the following: |  |
|  | Enthesitis (heel) |  |
|  | Positive family history |  |
|  | Psoriasis |  |
|  | Crohn’s disease, colitis ulcerosa |  |
|  | Urethritis/cervicitis or acute diarrhea within one month before arthritis |  |
|  | Buttock pain (alternating between right and left gluteal areas) |  |
|  | Sacroiliitis |  |

**Dougados M, van der Linden S, Juhlin R et al. The European Spondylarthropathy Study Group preliminary criteria for the classification of spondylarthropathy. Arthritis Rheum 34; 1218-27.*

Figure 3a:

| **ASAS Classification Criteria for Axial Spondyloarthritis (SpA)*** | | |
| --- | --- | --- |
| In patients with ≥3 months back pain and age at onset <45 years | | |
| Sacroiliitis on imaging**  plus ≥1 SpA feature | **OR** | HLA-B27  plus ≥2 SpA features |
|  | SpA-features: |  |
|  | - Inflammatory back pain |  |
|  | - Arthritis |  |
|  | - Enthesitis (heel) |  |
|  | - Uveitis |  |
|  | - Dactylitis |  |
|  | - Psoriasis |  |
|  | - Crohn’s/colitis |  |
|  | - Good response to NSAIDs |  |
|  | - Family history for SpA |  |
|  | - HLA-B27 |  |
|  | - Elevated CRP |  |

**Sacroiliitis on imaging

- Active (acute) inflammation on MRI highly suggestive of sacroiliitis associated with SpA
- Definite radiographic sacroiliitis according to the modified New York criteria

**Rudwaleit M, van der Heijde D, Landewe R et al. The development of Assessment of SpondyloArthritis international Society classification criteria for axial spondyloarthritis (part II): validation and final selection. Ann Rheum Dis 68; 777-83.*

Figure 3b:

| **ASAS Classification Criteria for Peripheral Spondyloarthritis (SpA)*** | | |
| --- | --- | --- |
| Arthritis or enthesitis or dactylitis  ***plus*** | | |
| ≥1 SpA feature | **OR** | ≥2 SpA features |
| - Uveitis |  | - Arthritis |
| - Psoriasis |  | - Enthesitis |
| - Crohn’s/colitis |  | - Dactylitis |
| - Preceding infection |  | - IBP (ever) |
| - HLA-B27 |  | - Family history for SpA |
| - Sacroiliitis on imaging |  |  |

Peripheral arthritis usually predominantly lower limbs and/or asymmetric arthritis

Enthesitis: clinically assessed

Dactylitis: clinically assessed

IBP: inflammatory back pain

**Rudwaleit M, van der Heijde D, Landewe R et al. The development of Assessment of SpondyloArthritis international Society classification criteria for axial spondyloarthritis (part II): validation and final selection. Ann Rheum Dis 68; 777-83.*

Figure 4:

| **Classification of Psoriatic Arthritis: CASPAR criteria***  ClASsification criteria for Psoriatic ARthritis | |
| --- | --- |
| To meet the CASPAR criteria for PsA, a patient must have inflammatory articular disease  (joint, bone, spine, or entheseal) and score ≥3 points based on these categories | |
|  | Points |
| 1. Evidence of psoriasis  - Current psoriasis - Personal history of psoriasis - Family history of psoriasis | - 2 or - 1 or - 1 |
| 1. Psoriatic nail dystrophy   Pitting, onycholysis, hyperkeratosis | - 1 |
| 1. Negative result for rheumatoid factor | - 1 |
| 1. Dactylitis  - Current swelling of an entire digit - History of dactylitis | - 1 or - 1 |
| 1. Radiologic evidence of juxta-articular new bone formation: ill-defined ossification near joint margins on plain x-rays of hand/foot | - 1 |

** Taylor W, Gladman D, Helliwell P, Marchesoni A, Mease P, Mielants H Classification criteria for psoriatic arthritis: development of new criteria from a large international study. Arthritis Rheum 54; 2665-73.*
