# Supplementary material for: The performance of different classification criteria sets for spondyloarthritis in the worldwide ASAS-COMOSPA study
Source: Arthritis Res Ther. 2017 May 16;19:96. doi: 10.1186/s13075-017-1281-5 (PMC5434574; doi:10.1186/s13075-017-1281-5)
Supplement: Supplementary file 2 — Ethical bodies that approved the study in the various centres. (DOCX 14 kb) [file 13075_2017_1281_MOESM2_ESM.docx]

Additional file 2: Ethical bodies that have approved the study in the various centers

Commissie Medische Ethiek, LUMC, Leiden

Ethisch comite, UZ Gent-trial bureau, Gent

Comité de protection des personnes Ile de France III, hopital cochin, france

Comité de etica de la investigacion de Cordoba, Consejera de Salud, Hospital Universitario Reina Sofia, Cordoba

Ethik-Kommission Arztekammer Westfalen-Lippe, Medizinischen Fakultat der Westfalischen Wilhelms-Universitat Munster, Munster

NRES Committee North East, Health Research Authority, Newcastle and North Tyneside, UK

Research Ethics Committee (REC), Faculty of Medicine, Cairo University

Comité d'Ethique pour la Recherche Biomedicale de Rabat

OHSU Research Integrity Office, Portland, USA

St. Luke's International Hospital Research Ethics Committee, Tokyo

Institutional Review Board, Chung Shan medical university hospital, Taiwan

Comité de Revision Institucional, Hospital Britanico, Buenos Aires

Erciyes Universitesi Klinik Arastirmalar Etik Kurulu Karar Formu, Kayseri, Turkey

NHG Domain Specific Review Board, National University Hospital, Singapore
